# Current insights in to the pathophysiology of Irritable Bowel Syndrome

**DOI:** 10.1186/1757-4749-2-3

**Published:** 2010-05-13

**Authors:** Theodoros Karantanos, Theofano Markoutsaki, Maria Gazouli, Nicholas P Anagnou, Dimitrios G Karamanolis

**Affiliations:** 1 Laboratory of Biology, University of Athens, School of Medicine and Biomedical Research Foundation of the Academy of Athens, Greece; 2 2nd Gastroenterology Department, Evangelismos Hospital, Athens, Greece

## Abstract

Irritable Bowel Syndrome (IBS) represents a functional disorder of gastrointestinal tract without the presence of an anatomic defect, in which abdominal pain is relieved with defecation and is associated with altered bowel habits.

IBS includes a wide range of symptoms while its pathophysiology is very complicated. Recent studies indicate that the most important mechanisms include visceral sensitivity, abnormal gut motility and autonomous nervous system dysfunction. The interactions between these three mechanisms make bowel's function susceptible to many exogenous and endogenous factors like gastrointestinal flora, feeding and psychosocial factors. Recent data indicate that according to the above mechanisms, the influence of genetic factors and polymorphisms of human DNA in the development of IBS is equally important.

## Introduction

IBS is a chronic continuous or remittent functional gastrointestinal disorder, characterized by abdominal pain, bloating and bowel disturbance [1]. The Rome Committee for the Classification of Functional Gastrointestinal Disorders has defined IBS on the basis of abdominal and bowel symptoms that occur with sufficient frequency in affected patients. More precisely, abdominal discomfort or pain must be observed for at least 3 days every month, for 3 months consecutive. The pain has two of the following three features:

1) Relief by defecation

2) Onset associated with a change in the frequency of stool

3) Onset associated with a change in the form of the stool

At least 1/3 of the general population presents with symptoms consistent with those of IBS. IBS seems to be more frequent on low socioeconomic groups, which may reflect unknown environmental factors, whereas these appear to be a modest decline in prevalence with advancing age [2].

Clinically there are two categories of IBS. In the first category, the prominent symptom is constipation, whereas in the second, patients document diarrhea, as a frequent manifestation of bowel dysfunction [3].

The etiology of IBS is most likely multifactorial. Several environmental factors, psychosocial stressors, gut flora alterations contribute to the pathophysiology of IBS, along with abnormal gastrointestinal motility and secretion and altered visceral perception. Today IBS is viewed upon as a disorder of dysregulation of the brain-gut axis, involving abnormal function in the enteric, autonomic and/or central nervous systems [4].

The purpose of this review is to discuss the most important pathophysiologic mechanisms that contribute to the genesis of IBS, as well as to document the known etiologic factors that have been correlated so far with the clinical manifestation of IBS.

## Pathophysiological Mechanisms

### Visceral Hypersensitivity

The latest data indicate that the main mechanism inducing abdominal pain is the visceral hypersensitivity [5]. The primary afferent neuron terminals of enteric nervous system (ENS) which are localized in submucosal tunica of gastrointestinal tract (Meissner plexus) and between smooth muscle fibers (Auerbach plexus) transmit stimuli to central nervous system (CNS) through sympathetic and parasympathetic autonomic nervous system (SANS and PANS). SNS transmits stimuli which are recognized as abdominal pain, whereas PNS transmits stimuli initiating a variety of reflexes. The pain stimuli through thalamus stimulate the cerebral cortex and permit the recognition of visceral pain. On the other hand, for the integration of visceral reflexes, the afferent stimuli through hypothalamus stimulate efferent neural fibers which through PNS stimulate or inhibit the contraction of smooth muscle fibers and the secretion of enterocytes in the gastrointestinal tract modifying the gut motility and secretion [5].

It is known that visceral sensitivity is regulated in many levels. Specifically this regulation is mediated at the level of enteric mucosa and submucosa, the level of spinal cord, the level of thalamus and the level of cerebral cortex.

#### Visceral sensitivity at the level of enteric mucosa and submucosa

The presence of an injury in enteric mucosa leads to the release of chemical mediators like K^+^, ATP and bradykinin but also inflammatory mediators like prostaglandin E_2 _(PGE_2_) [6]. These substances can directly stimulate the afferent neuron terminals but also can induce the release of algogenic substances (histamine, serotonin (5HT), nerve growth factor (NGF) and prostaglandins). This cascade leads to the amplification of the stimulus which represents the visceral pain [7].

There is particular interest about the interactions between afferent neuron terminals and mast cells. The release of substance P from the neuron terminals induces the production and release of histamine and NGF from mast cells. Histamine amplifies the release of substance P, whereas NGF seems to be implicated in neuron terminal's plasticity [8]. Recent data attribute the enhancement of neural sensitivity for algogenic stimuli to increased expression of sodium channels on primary afferent endings [8].

Some of the inflammatory mediators, which are released in enteric mucosa and submucosa seem to play a more spesific role. Serotonin (5HT), is believed to stimulate the primary afferent neuron terminals. Recent studies on pseudoaffective (cardiovascular) reflex responses to gut distension have suggested an action through a 5HT_3 _receptor subtype coupled to a sodium channel present on primary afferent endings. 5HT_3 _antagonists injected intravenously at low doses, potentiate visceral analgesic effects in response to gut distension in different rat models of abdominal pain [9].

It is known that bradykinin's signaling, influences visceral sensitivity in many ways [10]. Two bradykinin (BK) receptor types have been identified. There are studies that support that BK receptors are selectively upregulated during processes that follow some types of intestinal tissue injury and inflammation. Endogenous NGF released from mast cells under various stimuli may increase the primary afferent sensitivity to BK by upregulating BK receptors. Studies in experimental animals have shown that pharmacological agents which act as BK antagonists relieve the abdominal pain induced by intraperitoneal administration of acetic acid and urate crystals [11].

At the level of mucosa and submucosa, a variety of mediators like adenosine, tachykinin, calcitonin gene-related peptide (CGRP) and neurokinins participate in a cascade of events. Many C afferent fibers have "silent receptors" for neurokinins that can be sensitized by inflammatory processes in peripheral tissues. Generally, nerve remodeling occurring during inflammation can trigger chronic hypersensitivity in the submucosa and other intestinal structures [12]. These changes are complex, time dependent and related to the nature of inflammation. The acute phase of *Nippostrongylus brasiliensis *infection is associated with a 2.5-fold increase in nerve content of the tissues, chiefly as a result of axonal dilatation. During the recovery phase when mast cell proliferation is established, the mean cross sectional area of the nerve decreases but there is an increase in the diameter of small fibers.

#### Visceral sensitivity at the level of spinal cord

The primary afferent neuron terminals stimulated by algogenic stimuli release neurotransmitters, increasing the efficancy of synaptic transmission between primary afferents and dorsal horn neurons, a process, referred to as central sensitization, which involves specific pre- and postsynaptic receptors [11]. The mechanisms that underline central sensitization are not fully understood. In vivo and in vitro pharmacological studies implicate cooperation between substance P (SP) and N-methyl-D-aspartate (NMDA) [6]. Recent studies support the idea that NMDA receptors are implicated both at the level of spinal cord and peripherally- related to the sensitization of primary afferents. Substances that act as antagonists of these receptors can selectively decrease visceral hypersensitivity. The interaction of SP receptors with protein kinace C induces the phosphorylation of NMDA receptors, counteracting the magnesium block and allowing NMDA receptors to operate at a more negative potential [13]. In this way SP seems to amplify the sensitivity of algogenic signaling at this level as well. Recent data strongly suggest that SP and neurokinin NK_1 _receptors are crucial for the induction of central sensitization in rodents. However, the failure of NK_1_receptor antagonists in clinical trials for pain states indicates that another receptor may probably fulfill this action in humans.

CGRP is present in most splanchnic afferents, and CGRP immunoreactivity almost disappears from the gut after either splanchnic nerve section or treatment with the sensory neurotoxin capsaicin. Moreover, CGRP released at the spinal cord from central endings of primary afferents is important in the development of visceral hyperalgesia. Interestingly, peripherally released CGRP may modify sensory inputs, causing changes in blood flow, smooth muscle contractions, immune reaction and mast cell degranulation. The intravenous administration of the CGRP_1 _antagonist (h)-CGRP-(8-37) suppresses the abdominal cramps caused by intraperitoneal administration of acetic acid in awake rats [14].

Many studies pointed out that μ and κ opioid agonists lessen the nociceptive response to either peritoneal administration of irritants or intestinal distension. It seems also that κ agonists may act peripherally to prevent visceral pain and are more active in inflammatory conditions. It must be quoted that opioid receptors have been identified on both smooth muscle fibers and primary afferents localized in the gut [15]. These findings support that opioids are implicated in IBS development in a more complicated way and they influence not only the perception of the stimuli but stimuli per se as well.

Lastly, somatostatin and its receptors (SST_1 _and SST_2_) have also been identified on spinal cord and are probably related to the regulation of visceral pain, like GABA_A _and a_2 _adrenergic receptors.

#### Visceral sensitivity at the level of cerebral cortex and subcortical areas

There are very little facts in literature about regulation of visceral pain at the level of thalamus, limbic system and cortex. It is believed that serotoninergic pathways, inhibit neural impulses at the level of dorsal horn neurons. This effect, mainly through the action of GABA neurons, regulates the conduction of nociceptive stimuli to CNS [16]. It seems also that the interactions between serotoninergic pathways and limbic system are very important for the sensation of visceral pain. The role of limbic system is not clearly understood, but there are studies, which support the correlation between visceral hypersensitivity syndromes like IBS and emotional disorders like depression and bipolar disorders. Negative emotional conditions like fear and sorrow are related with abnormal sensory sensation and abdominal pain [17]. New studies with the use of functional magnetic resonance correlate the presentation of fearful expressions with stimulation of cortical and subcortical areas, which increase the sensation of visceral pain [18].

On the other side, stress is believed to modify the sensation of colon and rectum distention both in controls and IBS patients [19]. It seems that stress-induced release of CRF from subthalamus increases the production of mediators like histamine from mast cells. Interestingly, sensory stimuli which reach CNS stimulate subthalamus receptors leading to additional CRF release [20].

To summarise, the sensation of a visceral stimulus like colon distention or the presence of an irritative substance in the lumen is influenced by a variety of mediators at the level of enteric mucosa, spinal cord, thalamus and cerebral cortex. Inflammatory and non-inflammatory agents participate in the conduction and regulation of visceral stimuli, whereas neuropeptides seem to be released from inflammatory cells and neuron terminals.

### Abnormal Gut Motility and Secretory Disorders

#### Gut motility

The enteric nervous system (ENS), which is located in submucosa (Meisner plexus) and between smooth muscle fibers (Auerbach plexus) regulates the neuromuscular function of gastrointestinal (GI) tract. Sympathetic and parasympathetic autonomic nervous system (SANS and PANS) control the function of ENS, which is related to a variety of mediators and receptors, like serotonin and its receptors [21]. Serotonin is implicated in a variety of reflexes, which regulate the gut motility and secretory efficiency. These reflexes are integrated both at the level of enteric mucosa, through ENS and at the levels of spinal cord and subthalamus through PANS. It seems that secreted serotonin stimulates afferent terminals leading to a reflective gut peristalsis. In other words, the stimulation of afferent terminals directly modifies the gut motility [22].

The stimulated primary afferent neuron terminals synapse in the myenteric plexus (Auerbach plexus) with ascending and descending inter-neurons, thus inducing excitation and inhibition locally [23]. Ascending inter-neurons activate excitatory motor neurons by releasing substance P and acetylcholine (Ach) onto myocytes resulting in circular muscle contraction. Descending cholinergic neurons stimulate inhibitory motor neurons releasing nitric oxide (NO), vasoactive intestinal peptide (VIP) and adenosine triphosphate (ATP) leading to circular muscle relaxation. The resulting peristaltic reflex is largely responsible for the bolus movement from proximal to distal sites within GI tract [24].

Other studies evaluated the role of 5HT receptors in the peristaltic reflex and demonstrated the intricate involvement of CGRP. These investigators proposed that the primary intrinsic afferent involved in this reflex is a CGRP neuron activated by 5HT acting on 5HT_4 _receptors [25]. In human jejunum, rat and guinea pig colon, the 5HT_4 _agonist tegaserod stimulated ascending contraction and descending relaxation in vitro.

Activation of 5HT_3 _and 5HT_4 _receptors enhances gastrointestinal transit. Additionally, intrinsic afferents, utilizing 5HT_3 _receptors, may be involved in a reflex circuit within the gut that increases motility and intestinal secretions [26]. Antagonism of the 5HT3 receptors with ondansetron or alosetron delays colonic transit in healthy controls and in IBS patients with diarrhea as a predominant symptom [27].

New studies support that gut motility and defecation are regulated by psychical, somatic and immune stress [28]. The presence of a psychical and somatic stress like dehydration leads to the increased expression of hormone CRF in the paraventricular nucleus of hypothalamus [29]. The release of CRF from hypothalamic neurons stimulates parasympathetic efferent pathways, which increase gut motility. On the other hand, the intravenous injection of CRF to humans increased colon motility, with greater response to patients with IBS, which is an indication of the peripheral action of CRF [30].

#### 2.2.2 Secretion

The regulation of intestinal secretions is comparable to the regulation of gut motility. An initial stimulus stimulates the afferent terminals, which integrate reflexes, centrally through PANS and hypothalamus and peripherally through ENS. The efferent neuron terminals, which stimulate the intestinal secretion, are located in submucosa. The intestinal secretions are both directly stimulated by 5HT_4_receptors located in postsynaptic endings and indirectly by 5HT3 receptors, located at presynaptic endings. 5HT3 receptors are also located in the afferent neurons of PANS. These neuron cells transfer the sensory stimuli to hypothalamus for the integration of the reflex [31]. Substances, which are locally released from mast cells at the mucosa level, are also responsible for the reflective intestinal secretion. Besides serotonin, VIP and substance P are also important.

### Autonomic Nervous System Dysfunction

It is known that autonomic nervous system (ANS) regulates visceral sensitivity and modulates and coordinates GI motility and secretion [32]. New studies support that sections of ANS are implicated in the immunological regulation and inflammatory reaction at the level of enteric mucosa [33].

It is also reported that most of IBS symptoms are directly related to specific abnormalities of ANS. It seems that the main characteristic of IBS patients is the increased activity of SANS and the decreased activity of PANS [34]. There are differences between patients with diarrhea and constipation as predominant symptoms and between men and women. It is believed that vagal dysfunction is associated with constipation as a predominant symptom whereas adrenergic sympathetic dysfunction is associated with diarrhea as a predominant symptom [35]. Other studies reported that IBS diarrhea-predominant patients were shown to have cortisol hyper-responsiveness unlike that of constipation-predominant IBS patients and controls [36]. Robert et al. [37] and Tillish et al. [38], on the other hand, observed elevated sympathetic dominance and vagal withdrawal during non-REM and REM sleep in diarrhea-predominant IBS patients, but not in those with an alternating type of IBS. However, constipation-predominant IBS patients could not be distinguished from diarrhea-predominant IBS patients or alternating type IBS with regard to autonomic nervous system. It is reported that there might be a continuum of autonomic dysfunction among these symptom-specific subgroups [39].

Concerning sex-specific alterations in autonomic function among IBS patients, it is believed that there are differences in visceral sensitivity both at the level of mucosa, and at the level of central pain sensation [40]. Recent evidence has confirmed that sex differences exist among IBS patients in term of colonic transit response to pharmacological treatment [41]. These observations may justify the different IBS incidence between male and female. It is also found that male IBS patients had significantly higher sympathovagal balance than healthy male controls, whereas no differences were noted between IBS females and female controls. However, differences in autonomic nervous system responses to specific stimuli may also play a role in producing sex-based variations in IBS symptom patterns and in differential responses to some pharmacological agents [42].

Increased SNS activity and decreased PNS activity are the most frequent noted signs in IBS patients. We often observed cutaneous hyperalgia in IBS patients. Mayer et al. [43] reported that patients with IBS also exhibited a number of extraintestinal manifestations such as migraine headache, back pain, heartburn, dyspareunia and muscle pain, consistent with the central hyperalgesic mechanism.

## Etiologic Factors

### Small Intestine Bacterial Overgrowth Syndrome

About 65-84% of IBS patients present with small intestine bacterial overgrowth, that is, presence of more than 10^5 ^cfu/ml of bacteria, resembles the bacterial composition of the colon, in the proximal small bowel [44]. The colonization of gastrointestinal tract differs according to the type of bacteria. Particularly, bacteria from the upper respiratory tract and anaerobic bacteria from oral cavity via the slobber swallowing, colonize the upper gastrointestinal tract with a density of < 10^5 ^cfu/ml. The stomach is colonized by bacteria with a density of < 10^3 ^cfu/ml because the acidic pH does not permit the colonization and proliferation of bacteria coming from the oral cavity. In the small intestine, there is a progressive colonization of gram positive aerobic and facultative anaerobic bacteria, like staphylococcus, streptococcus, lactobacillus and some types of fungi. The presence of gram-negative and anaerobic bacteria in the proximal jejunum is abnormal. Strains of *Prevotella disiens *and *P. divia *are recognized in the small intestine. Normally, small intestinal microflora is believed to be exogenous and not endogenous [45]. Anaerobic strains and mainly *Bacteroides fragilis *are usually restricted in the distal jejunum and large intestine (Table 1). The ecology of small intestine's content is constant due to normal bowel motility, acidic pH and IgA secretion from small intestine mucosa.

**Table 1:** Microbiologic Ecology of the Normal Human Intestinal Tract

Oral cavity	*Staphylococcus epidermidis* *Streptococcus mitis* *Streptococcus salivarius* *Streptococcus mutans* *Lactobacillus spp*.
Stomach	*Helicobacter pylori* *Lactobacillus spp*.
Small bowel	*Bacteroides fragilis* *Prevotella disiens* *Prevotella divia* *Enterococcus spp* *Lactobacillus spp*.
Colon	*Bacteroides fragilis* *Enterococcus faecalis* *Bifidobacterium spp* *Peptostreptococcus spp* *Lactobacillus spp*. *Clostridium spp*. *Escherichia coli*

It has been documented that in pathological cases of small intestine bacterial overgrowth, there are excessive bacterial counts in the proximal small bowel, commonly with bacterial species including *Streptococci*, *Bacteroides*, *Escherichia*, and *Lactobacilli*.

Bacterial overgrowth implies abnormal colonization of the upper gastrointestinal tract, arising from failure of specific defense mechanisms restricting colonization under physiological conditions. These defense mechanisms are the gastric acid barrier and the intestinal clearance. *H. pylori *induced-gastritis is the main cause of acquired failure of the gastric acid barrier. Failure of intestinal clearance may come as a result of impaired intestinal persistalsis, in case of myopathic, neuropathic, autoimmune, infectious, metabolic, endocrine or neoplastic diseases. Anatomical abnormalities that alter luminal flow may as well cause failure of intestinal clearance. This may be the result of gastrointestinal surgery, intestinal diverticula, or fistula [46].

The bacterial content of oral cavity, host's general condition, immunological disorders and bad nutrition play important role in the development of bacterial overgrowth syndrome.

### Post Infectious Irritable Bowel Syndrome

Post infectious IBS (PI IBS) represents a subtype of IBS. It affects 6-17% of IBS patients, who had undergone a previous episode of infectious gastroenteritis [47]. While most patients rapidly recover from bacterial gastroenteritis, about a quarter show persistent disturbance of bowel habit at 6 months and most commonly increased stool frequency. A smaller number develop persistent symptoms that meet the Rome III criteria. Clinical features include bloating, loose and watery stools, urgency for defacation and the passage of mucus per rectum [48].

There are indications that an episode of acute gastroenteritis is capable to induce small intestine sensitization and symptoms of IBS, only if there are other factors mainly psychosocial, which can stimulate through psychical, neuronal and endocrinal mechanisms, the presence of mast cells and other inflammatory cells in the gastrointestinal tract [49].

PI IBS has been reported after *Campylobacter*, *Salmonella *and *Shigella *infections [50]. Those patients, who later on develop IBS, show increased numbers of enterochromaffin (EC) cells and lymphocyte cell counts at 3 months compared to those who do not develop IBS. Interleukin-1β (IL-1β) mRNA levels are increased in the mucosa of those who develop PI IBS and show also increased gut permeability. Recent studies suggest an increase in peripheral blood mononuclear cell cytokine production in unselected patients, an abnormality that may be ameliorated by probiotic treatment. Recovery from PI IBS may be slow, with approximately 50% of patients manifesting symptoms at 5 years.

One measure to estimate the probability to develop PI IBS, is the duration of the initial diarrheal illness. Diarrhea lasting more that 3 weeks gives a relative risk of 11.4 compared with diarrhea lasting less than 7 days. Changes in the epithelium, during the healing process, are also predictive of the development of PI IBS. Age above 60 gives a protective effect. It seems that older subjects have fewer immunocytes in their rectal mucosa and may be less reactive to infection. Depression and the presence of adverse life events double the relative risk of persistent symptoms [51].

### Diet

Acute hypersensitivity reactions are rare causes of IBS. Patients often present with atopic conditions, such as eczema, asthma, angioedema, while they respond well to elimination diets [52]. Those hypersensitivity reactions are mediated by degranulation of mast cells.

The degranulation of mast cells leads to the production of local and systemic mediators, such as leucotriens LTC4 and histamine, which act upon adjacent smooth muscle cells and nerve endings.

Lactose intolerance, as well as intolerance to sorbitol or fructose, has been implicated in IBS. It is likely that the specific enzyme deficiency is not the cause of IBS, but that the hypersensitive guts of patients with IBS show exaggerated responses to the gaseous and fluid distention caused by incomplete absorption of carbohydrate.

### Psychical Factors

The correlation between emotion and gut motility has now been established. Many physiology studies have shown that anger is closely associated with enhanced contractile activity in the sigmoid-rectum area, whereas reduced motility is documented in case of fear. Anxiety can induce rapid small bowel transit and enhanced stool frequency. Moreover, depression is associated with delayed small bowel and colonic transit.

The effects of emotion on gastrointestinal function are mediated by the autonomic nervous system. The normal physiologic response to acute stress involves the linked activation of the hypothalamo-pituitary axis and the sympathetic nervous system. The brain may influence the transmission of nociceptive information from the gut and the activation of visceral reflexes through descending inhibitory and excitatory pathways that terminate within the dorsal horn at the secondary sensory neuron.

Common characteristics of IBS patients are the pathologic gradation of visceral perception, the endogenous pain facilitation and the reduced threshold for pain. Abuse history is common in their past, exaggerated conscientiousness, perfectionism and neuroticism have been detected among their personality features [53].

## Genetic Predisposing

There is growing evidence regarding the genetic contribution in the syndrome [54]. The genetic base of IBS is supported by the fact that the syndrome has characteristics similar to multifactorial and multigenic disorders, such as the different incidence in different geographic areas [55].

The evaluation of genetic influence is based on familial aggregation, twin studies and genetic epidemiological studies focusing on gene polymorphisms [55].

### Familial Aggregation

Although the family clustering of IBS has been noticed in medical practice for several years, Whorwell et al. [56] found that 33% of patients with IBS reported a family history of IBS compared with only 2% of the control group. Newer studies showed relation between having a first degree relative with bowel problems and presenting with IBS, concluding that these results may represent exposure to similar environmental factors [57], although some limitations of these studies included the fact that only abdominal symptoms in first degree relatives were assessed and that the IBS diagnosis was not confirmed by a specialist.

### Twin Studies

An Australian study by Morris-Yates et al. [58], showed a higher concordance rate for IBS in monozygotic twins than in dizygotic twins (33.3% vs 13.3%). Interestingly, an American study conducted in 2001 showed that the concordance rate in monozygotic twins was twice as high as that in dizygotic twins (17.2% vs 8.4%) [59]. However, the number of dizygotic twins with IBS who have mothers with IBS was greater than the number of dizygotic twins with IBS who have co-twins with IBS; data also showed that having a mother or a father with IBS are both independent predictors of irritable bowel status and are stronger predictors than having a twin with IBS. These results support also that social learning has an important influence [59]. However a Norwegian study published in 2006 showed a concordance for IBS significantly greater in monozygotic than in dizygotic twins. The same study, showed, that low birth weight also influences significantly the development of IBS [60]. Finally, another twin study published in 2007 concluded that the genetic factors are involved in IBS, possibly by the hereditability of anxiety and depression [61].

### Gene Polymorphisms

The delineation of the regulation of the fut-brain axis at the level of sensation and motility by mediators like 5HT, cholecystokinin (CCK) and substance P, has lead to the investigation of a variety of polymorphisms. Gene polymorphisms involve the serotonergic and adrenergic systems and genes encoding proteins with immunomodulatory and/or neuromodulatory features [62]. One candidate gene is the serotonin transporter gene (SERT). The serotonin transporter protein is responsible for reuptake of serotonin from the synaptic cleft. Within this gene, there is a 44 bp insertion/deletion of repeat elements in the promoter region. This polymorphism results in a long (*l*), and a sort (*s*) allele. The *s *allele is associated with lower transcriptional efficiency and therefore lower serotonin transporter expression, and decreased cellular uptake of serotonin. Recent studies showed relation between the presence of *l/l *genotype and IBS with constipation as the predominant symptom and decreased response to tegaserod [63]. Other studies revealed that the presence of the *s/s *genotype is related to IBS with diarrhea as the predominant symptom, particularly in women [63]. A recent Indian study showed that the presence of the *s/s *genotype is related to IBS with constipation as the predominant symptom [64].

Association studies of polymorphisms in genes encoding CCK [65], adrenergic receptors [66] and cytokines like TNF-a and IL-10 investigating the relation between IBS and inflammation [67] have been also reported.

Finally, IBS is a multifactorial functional disorder which is related to genetic, environmental and psychological factors. Due to the important influence of environmental and psychological factors in the development of IBS many studies are required to confirm the genetic base of the syndrome.

## Competing interests

The authors declare that they have no competing interests.

## Authors' contributions

TK and TM participated in the writing of the manuscript. MG, NPA and DGK edit the manuscript. All authors read and approved the final manuscript
